# Expanding the scope and visibility of ambulatory stewardship programs with novel coronavirus disease 2019 (COVID-19) therapeutics

**DOI:** 10.1017/ash.2022.52

**Published:** 2022-04-29

**Authors:** Hongkai Bao, Yi Guo, Mei H. Chang, Terrence McSweeney, Austin M. Golia, Kelsie Cowman, Rachel Bartash, Brenda I. Anosike, Priya Nori

**Affiliations:** 1 Department of Pharmacy, Montefiore Health System, Albert Einstein College of Medicine, Bronx, New York; 2 Network Performance Group, Montefiore Health System, Bronx, New York; 3 Division of Infectious Diseases, Department of Medicine, Montefiore Health System, Albert Einstein College of Medicine, Bronx, New York; 4 Division of Infectious Diseases, Children’s Hospital at Montefiore, Montefiore Health System, Albert Einstein College of Medicine, Bronx, New York

## Abstract

Antimicrobial stewardship programs (ASPs) can be expanded to the outpatient setting to serve as a first line of defense against coronavirus disease 19 (COVID-19) hospitalizations and to reduce the burden on emergency departments and acute-care hospitals. Given the numerous emergency use authorizations of monoclonal antibodies and oral antivirals, ASPs possess the expertise and leadership to direct ambulatory COVID-19 initiatives and transform it into a predominantly outpatient illness. In this review, we summarize the critical role and benefits of an ASP-championed ambulatory COVID-19 therapeutics program.

Maximizing therapeutics targeting the highest-risk coronavirus disease 2019 (COVID-19) outpatients can alleviate the burden on emergency departments (EDs), acute-care hospitals, and an exhausted frontline workforce. Global emphasis on vaccination, early testing, and antiviral treatments are public health priorities in the response to endure future pandemic surges and emerging severe acute respiratory syndrome coronavirus-2 (SARS-CoV-2) variants. Ensuring adequate supply to match disease burden can transform COVID-19 into a predominantly outpatient illness.^1,2^ Since November 2020, the Food and Drug Administration (FDA) has issued multiple emergency use authorizations (EUAs) for novel outpatient SARS-CoV-2 antiviral therapies. These include monoclonal antibodies (mAbs) for treatment and prophylaxis as well as oral antiviral therapies. In response to the surge of the (omicron or B.1.1.529) variant, the FDA has expanded remdesivir approval for outpatients, further broadening the outpatient armamentarium.

A myriad of examples of inpatient antimicrobial stewardship program (ASPs) contributions to the pandemic response exist.^3,4^ However, expansion of stewardship efforts to the outpatient setting can serve as a “first line of defense,” preventing COVID-19 hospitalizations and enabling inpatient stewardship to focus on preventing excess antibiotic use and resistance. Our hospital ASP, like others, rapidly developed systemwide, dynamic, outpatient antiviral treatment pathways with the primary goals of alleviating the burden on our frontline workforce and preventing severe outcomes in a highly vulnerable population. Antimicrobial stewards are poised to lead these efforts, provided the necessary infrastructure is in place.^3,4^

## Monoclonal antibodies

Beginning November 2020, the FDA has issued multiple EUAs for mAbs administered as monotherapy or combination therapy. These mAbs act by binding to the receptor binding domain of the SARS-CoV-2 spike protein, preventing attachment to the host angiotensin converting enzyme-2 (ACE-2) receptor and thus blocking initial stages of infection. When administered to high-risk adult and pediatric patients (aged ≥12 years and weighing at least 40 kg) early in disease, mAbs can reduce hospitalizations, deaths, symptom duration, and viral loads (Table 1).^5,6^ Clinical trial outcomes have been reinforced by numerous real-world studies.^7–11^ EUA status ensures that medications are free to patients and facilities; however, facilities can charge for televisits, nursing services, and administration fees. Therefore, both patients and healthcare systems benefit from operationalizing mAb programs.

**Table 1.** Outpatient COVID-19 Therapeutics Currently Recommended by National Guidelines^23,29^

Drug	Target Population	Mechanism	Dosage	Time to Start from Symptom Onset	Risk Reduction for Hospitalization or Death (Study)	Comments
**Small-molecule antivirals**						
Nirmatrelvir/ ritonavir (Paxlovid)	High-risk patients who qualify (≥12 years, ≥40 kg)	Blocks activity of SARS-CoV-2 3CL protease enzyme (proteolysis); ritonavir used as pharmacokinetic booster	Two 150-mg tablets of nirmatrelvir with one 100-mg tablet of ritonavir twice daily for 5 days; copackaged	≤5 d	89% (EPIC-HR)	• Complex drug–drug interactions • Renal/hepatic dose adjustments • Pregnant and lactating patients excluded • Common side effects: dysgeusia, diarrhea, hypertension, myalgia
Remdesivir	High-risk patients who qualify (all age groups, must weigh ≥3.5 kg) Preferred when local variant prevalence is high or when contraindications or availability precludes receipt of other agents	Direct-acting nucleotide prodrug inhibitor of SARS-CoV-2 RNA-dependent RNA polymerase	200-mg IV on day 1, followed by 100-mg IV on days 2–3 Pediatric patients aged <12 y or <40 kg must use weight-based dosing	≤7 d	87% (PINETREE)	• FDA approved for hospitalized and nonhospitalized patients ≥12 y, ≥40 kg • EUA for hospitalized and nonhospitalized pediatric patients <12 y, or underweight patients • Due to unique mechanism of action, not impacted by SARS-CoV-2 variants • Requires infusion center, logistically challenging • Transaminase elevations
Molnupiravir	Patients not qualifying for other therapeutics (≥18 years)	Potent ribonucleoside analog that inhibits the replication of SARS-CoV-2 by introducing mutations	Four 200-mg capsules twice daily for 5 days	≤5 d	30% (MOVe-OUT)	• Low efficacy compared with other treatments (should only be used if all other options are unavailable) • Fetal toxicity in animal studies • Common side effects: diarrhea, nausea, dizziness
**Monoclonal antibodies**^a^						
Sotrovimab^b^	Highest-risk outpatients or those contraindicated for Paxlovid (≥12 years, ≥40 kg)	Attaches to SARS-CoV-2 spike protein receptor binding domain to prevent attachment to host cells via ACE-2 receptor	Single 500-mg dose IV over 15–30 minutes	≤7 d	79% (COMET-ICE)	• Requires access to infusion center or home infusion program, or infusion capacity in long term care facilities • Preferred for severely immunocompromised (eg, transplant patients on tacrolimus) • Hypersensitivity reactions can occur
Bebtelovimab	Similar to sotrovimab, preferred if prevalence of resistant variants [eg, (omicron) subvariant BA.2] in community is high	Similar to other mAb therapies	Single 175-mg dose IV over ≥30 seconds	≤7 d	Insufficient data, extrapolated from other mAbs	• Requires access to infusion center or home infusion program, or infusion capacity in long term care facilities • Preferred for severely immunocompromised (eg, transplant patients on tacrolimus) • Effective against (omicron) subvariants (eg, BA.2) • Hypersensitivity and infusion reactions can occur
Tixagevimab/ cilgavimab (EVUSHELD) for pre-exposure prophylaxis only	Severely immunocompromised patients unable to mount vaccine response, or those unable to receive vaccination (≥12 years, ≥40 kg)	Similar to other mAb therapies, prolonged half-life	Single injections of 300-mg IM tixagevimab and 300-mg IM cilgavimab	N/A	77%^c^ (PROVENT)	• Requires infusion center or home infusion program • Potential decreased efficacy versus (omicron) variant prompted doubling dose • Long half-life and efficacy up to 6 months • Administer ≥2 weeks after COVID-19 vaccination • Effective against (omicron) subvariant BA.2 • Hypersensitivity reactions can occur

Note. mAb, monoclonal antibody; EUA, emergency use authorization; FDA, Food and Drug Administration; N/A, not available.

a Bamlanivimab, bamlanivimab-etesevimab, and casirivimab-imdevimab are previously authorized monoclonal antibodies that are no longer in use and have been omitted due to inefficacy against currently circulating SARS-CoV-2 variants.

b Due to the increasing prevalence of (omicron) subvariant BA.2, authorization for sotrovimab has been rescinded in certain territories and may be rescinded nationally.

c Risk reduction for preventing symptomatic COVID-19.

In late 2020, bamlanivimab was authorized^12^ followed by casirivimab and imdevimab.^13^ Subsequently, etesevimab was added to bamlanivimab to provide greater efficacy against circulating variants and the bamlanivimab monotherapy EUA was revoked.^14^ Sotrovimab was the mainstay of treatment during surge of the (omicron) variant^15^; however, due to increasing prevalence of the (omicron) subvariant BA.2 and concerns about reduced in vitro SARS-CoV-2 neutralization,^20^ bebtelovimab EUA was rapidly issued on February 11, 2022, even though the surge was already subsiding in several states (Table 1).^16,17^

Immunocompromised populations have been disproportionately affected by severe outcomes and excess mortality from COVID-19.^18^ Due to lower antibody responses to vaccination,^19^ these patients remain at significant risk, especially as the (omicron) surge subsides and public health measures are significantly relaxed. Therefore, the availability of tixagevimab-cilgavimab upon EUA for pre-exposure prophylaxis in December 2021 was considered a major advance. Tixagevimab-cilgavimab has a half-life of nearly 3 months, providing potentially 6 months of protection.^20^ To date, tixagevimab-cilgavimab is the only agent authorized for this indication.

## Small molecule antivirals

Several mAb therapies have been rendered ineffective by SARS-CoV-2 variants over time; therefore, small-molecule antivirals, which likely retain activity against variants, will have an essential role in contending with future surges.

Remdesivir received FDA approval first for hospitalized patients with COVID-19, demonstrating hastened clinical improvement compared to placebo.^21^ In January 2022, FDA approval expanded to ambulatory adult and pediatric populations.^22^ Compared to placebo, 3 days of remdesivir decreased the risk of hospitalization by 87% (Table 1), leading to official guideline endorsement and cementing its role in the outpatient COVID-19 armamentarium.^23,24^ FDA approval ensures that reimbursement to infusion programs is not an obstacle; however, securing 3 consecutive days of intravenous remdesivir therapy, whether through home infusion or dedicated infusion centers, is challenging, especially for infectious patients who may expose others in the process. However, given its mechanism of action, it may serve a role during future surges dominated by variants resistant to antibody therapies.

Molnupiravir, an oral antiviral targeting SARS-CoV-2 RNA replication and inducing viral mutagenesis, received EUA in December 2021 for treatment of mild-to-moderate COVID-19 in patients aged ≥18 years at high risk for progression, presenting within 5 days of symptom onset with a confirmatory direct viral test. In clinical trials, molnupiravir reduced the risk of hospitalization or death compared to placebo by 30% (Table 1).^25^ However, it is associated with potential teratogenicity, and its mechanism of action may hasten development of viral resistance.^26^ Consequently, the FDA recommends against its use during pregnancy and advises individuals of child-bearing potential to use contraception for an extended period following completion. Therefore, providers should prescribe molnupiravir for the appropriate patient when other agents are unavailable or are contraindicated.

Nirmatrelvir copackaged with ritonavir, also received EUA in December 2021 for similar indications. However, nirmatrelvir-ritonavir demonstrated significantly higher efficacy compared to molnupiravir, with an 88% reduction in COVID-19–related hospitalization or death in clinical trials (Table 1).^27^ However, coformulation with ritonavir potentiates numerous drug–drug interactions with concomitant drugs such as statins, calcineurin inhibitors, and psychiatric medications. Despite the convenience of a 5-day, at-home, oral antiviral regimen, provider concerns about drug interactions present a significant hurdle to its uptake.

## The critical role of stewardship in outpatient COVID-19 therapeutic programs

An ASP-led “command center” has numerous advantages for optimizing outpatient COVID-19 treatment and prophylaxis. First, ASPs have vast experience in implementing preauthorization paradigms in the inpatient setting. Second, aligning with the Centers for Disease Control and Prevention’s Core Elements of Outpatient Antimicrobial Stewardship, ASPs possess accountability, drug expertise, and the ability to track and report metrics after implementation of therapeutic interventions.^28^ Third, ASP-created protocols can be disseminated to distal locations across the healthcare network such as private practices, urgent-care centers, hemodialysis centers, and school-based clinics. Finally, ASPs can establish complex treatment programs, garner regional repute, troubleshoot adverse events and logistical challenges (eg, IV bag or tubing shortages), master rapidly involving science, and apply clinical expertise to provide optimal care for patients. The physician ASP-lead can ensure the support from institutional leadership for human, financial, and information technology resources, ensure accountability for meeting goals and targets, and help drive the research agenda of the program. Pharmacists, with complementary skills to physicians, expertly communicate medical information to patients and frontline nurses, maintain inventory, coordinate compounding and delivery of medications, report adverse medication events, and develop electronic decision support and guidance for optimal use.

With assistance from national guidelines, ASPs can develop systemwide protocols to integrate all available treatments and assist providers in selecting the most appropriate therapy for each patient (ie, mAbs, remdesivir, molnupiravir, or nirmatrelvir-ritonavir) when considering (1) drug availability, (2) host factors, and (3) feasibility and logistics.^23,29^

## Barriers to implementation and proposed solutions

**Fig. 1.**
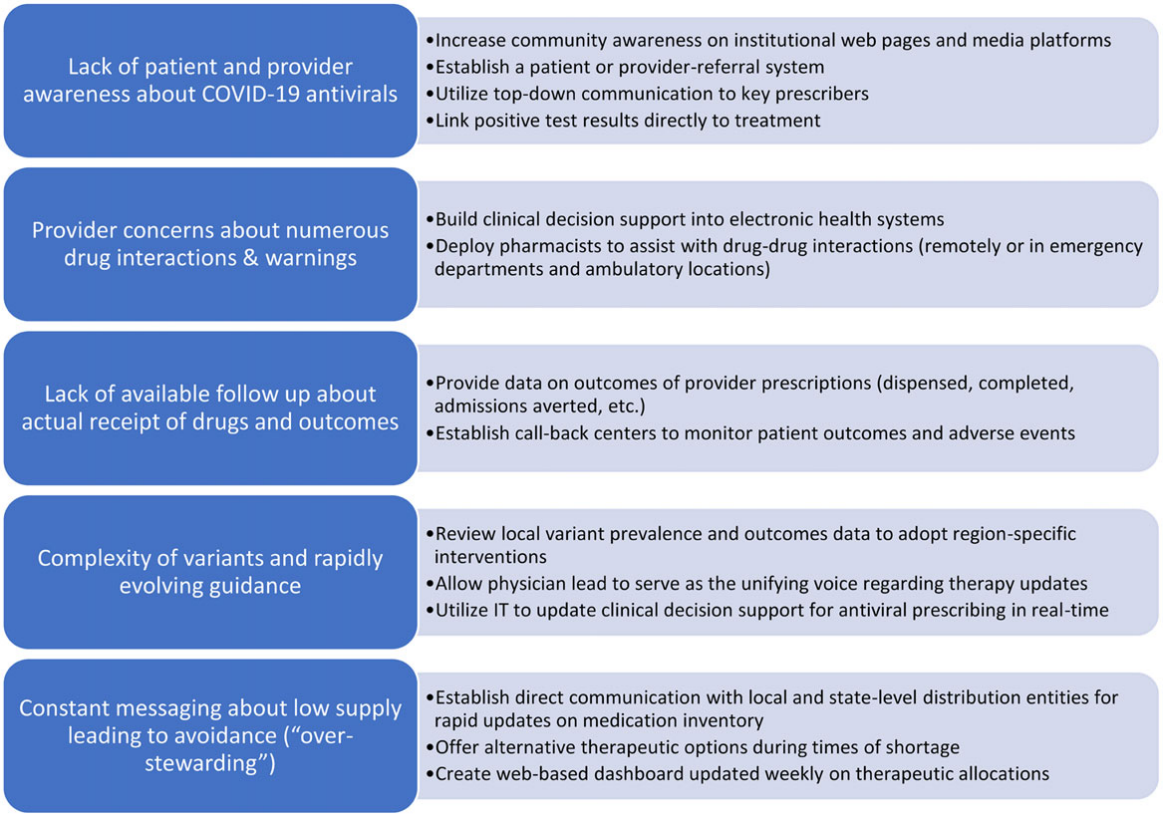
Recommendations by antimicrobial stewardship programs to overcome barriers to outpatient COVID-19 antiviral prescribing.

Although ASPs are equipped to lead ambulatory COVID-19 treatment efforts, numerous barriers to implementation must be overcome (Fig. 1). Connecting patients to care and administering treatments during the optimal window of efficacy are both major challenges. EUAs for mAbs require administration within 7–10 days of symptom onset,^13,14,30^ and real-world studies indicate a positive correlation between time to treatment and risk of hospitalization.^31^ Likewise, clinical trials indicate maximal efficacy when oral antiviral agents are administered within 5 days of illness onset.^25,27^ To ensure timely and equitable access, we developed parallel treatment pathways in an ambulatory infusion center and multiple EDs to provide access 24 hours per day, 7 days per week.^32^ Other centers have utilized mobile infusion vans or home infusion programs to expand access.^33,34^ It has also been extremely beneficial to establish a patient and provider referral system directly to the infusion staff so that patients are connected to care in real time.

The rollout of nirmatrelvir-ritonavir was slower than anticipated due to concerns of numerous drug–drug interactions and warnings. Fortunately, ASP pharmacists are well-equipped to handle drug–drug interactions, to ensure appropriate renal-dose adjustments, and to build clinical decision support into electronic order sets. Moreover, given the initial high demand of oral antivirals, our ASP liaised with local health departments and state-designated community pharmacies to track supply and communicate updates to prescribers. We also developed feedback reports to help increase provider uptake by displaying prescription volume head-to-head among all ambulatory locations.

Another implementation obstacle to outpatient COVID-19 antiviral implementation was the pace at which new EUAs and guidelines were issued and revised based on new data, which were often released as non–peer-reviewed, preprint studies.^35–37^ ID specialists within ASPs have command of the pathophysiology of COVID-19 and pharmacology of novel therapeutics. This example reveals the importance of data-driven, ASP-led therapeutic decisions. The ι (iota or B.1.526) variant, which spread throughout New York state in early 2021, possessed the E484K mutation,^38^ rendering bamlanivimab monotherapy ineffective but not casirivimab-imdevimab.^12–14^ The incidence of the ι (iota) variant peaked at 45% of all COVID-19 cases in New York state in March–April of 2021,^39^ but the distribution and FDA EUA for ineffective mAbs was not rescinded until June 2021.^40^ This example highlights the need for adopting region-specific recommendations that may precede EUA revisions or national guideline updates.^41^

Supply-and-demand mismatch of novel therapeutics has challenged healthcare systems throughout the pandemic. The most pronounced example occurred during the surge of the (omicron) variant, when the only effective mAbs were sotrovimab and later bebtelovimab.^16,17,42^ Several programs, including ours, were forced to pause operations until sotrovimab became available and to cease treatment with casirivimab-imdevimab despite high patient and provider demand.^43^ An overwhelming surge of new cases meant that decisions were required in real time prior to confirmation of the predominance of the (omicron) variant by public health authorities. Given extremely limited supply, programs were pushed to prioritize patients at highest risk of death, such as severely immunocompromised or unvaccinated patients with multiple comorbidities.^44^

Implementation of tixagevimab-cilgavimab for pre-exposure prophylaxis was met with multiple challenges: (1) supply limitations and expected high demand required programs to enact strict use criteria, (2) administration could not take place in usual mAb infusion locations containing COVID-infected patients, and (3) provider acceptance was hindered by emerging data questioning its efficacy against the (omicron) variant.^42,45^ In response, the FDA revised the EUA in February 2022 to recommend a doubling of the prior dose, requiring programs to contact treated patients to return for a supplemental dose.^46^ Several weeks following the initial EUA, the Centers for Disease Control and Prevention formally endorsed a fourth mRNA vaccination for immunocompromised patients,^47^ further delaying tixagevimab-cilgavimab administration in eligible patients. The ideal timing and role of this agent (eg, outpatient vs inpatient administration, timing after infection or treatment with other mAbs, timing of subsequent vaccination) remains to be elucidated. Not surprisingly, uptake has been suboptimal and rollout has been uneven nationally.^48^

As we and other ASPs have learned in times of crisis, undertaking major therapeutic decisions impacting potentially thousands of vulnerable patients requires a multidisciplinary approach with thoughtful input from ID physicians, pharmacists, nursing champions, hospital leadership, and medical ethicists.

## Pediatric experience

Immunocompromised children or those with medical comorbidities are at an increased risk for severe disease.^49^ Multisystem inflammatory syndrome is significantly more common in pediatric patients. Unfortunately, no pediatric-specific, placebo-controlled, randomized clinical trials of antiviral therapies have been conducted to date. Given the limited pediatric treatment options, provider practices are highly variable. MAb therapies and oral nirmatrelvir-ritonavir are authorized only for patients aged ≥12 years and weighing at least 40 kg. Remdesivir can be administered to pediatric patients weighing at least 3.5 kg, but outpatient administration is challenging, as described previously. Tixagevimab-cilgavimab is authorized for pre-exposure prophylaxis in select pediatric patients, but lack of data remains an obstacle. Vaccines remain a critical preventative strategy for severe COVID-19 disease and hospitalization, but uptake in school-aged children has been suboptimal and authorization for younger children remains delayed.

ASPs have been essential in pediatric COVID-19 treatment evaluations, carefully weighing known risks and benefits against unknowns. ASP-developed, pediatric-specific, clinical algorithms can help ensure timely and equitable access to outpatient therapies, though more high-quality studies and tools are needed to improve risk-stratification and to optimize outcomes.

## Tracking and reporting

Tracking and reporting antimicrobial utilization is an essential stewardship performance element endorsed by the Centers for Disease Control and Prevention and regulatory agencies, which can also be applied to COVID-19 therapeutics. Real-time tracking of mAb referral volume portended the surges of the δ (delta or B.1.617.2) and (omicron) variants at our medical center. Monitoring weekly mAb utilization is required for reporting to local, state, and federal entities, and it can help navigate supply shortages and enable modification of institutional guidelines to prioritize the highest-risk patients. Alternatively, if utilization is lower than expected, intensified community outreach and physician education are needed to increase awareness.

MAb utilization metrics can help programs allocate staff and resources appropriately and identify potential barriers. For example, early data revealed a gap in mAb administrations in the ED overnight and on weekends, leading to a backlog at our infusion center. Acknowledging the potential of a fully operational mAb program in our EDs, hospital leadership provided infusion resources available 24 hours per day, 7 days per week. We recommend that programs create and disseminate recurring mAb stakeholder reports with referral volumes, adult and pediatric administrations (by location, date, and time), adverse reactions, posttreatment ED presentations, admissions, and deaths. Weekly reports to executive leadership have the potential to greatly increase the visibility of mAb programs and to establish the role of the ASP role as the standard bearer of the health system. Closely tracking 14-day and 30-day posttreatment hospitalizations may also inform therapeutic protocol updates and/or signal the arrival of new variants.^41^

## Electronic builds and decision support

A core function of ASPs is the development of electronic builds for antimicrobial orders with clinical decision support. Several ASPs have developed an electronic mAb program referral order that includes the following information: (1) date of SARS-CoV-2 positive test, (2) COVID-19 vaccination history, (3) date of symptom onset, (4) description of symptoms, and (5) risk factors for progression to severe disease. The clinical information provided within the referral order can greatly expedite the workflow of infusion teams and help determine the most appropriate treatment for each patient (eg, mAbs vs oral antivirals).

Additionally, creating a best-practice alert for ED providers that flags patients who present to EDs with positive SARS-CoV-2 tests can prompt them to assess eligibility for COVID-19 outpatient therapeutics. Restricting mAb ordering access to only outpatient sectors and EDs can discourage EUA-nonconcordant prescribing for hospitalized patients.

When novel oral antivirals received EUA, we expedited the development of ambulatory order sets with decision support containing patient demographics, drug–drug interactions, warnings, renal-dose adjustments, and indications for use. Feedback from providers has suggested that automatic alerts in the electronic health record for relevant drug–drug interactions between nirmatrelvir-ritonavir and patients’ home medications help reduce prescribing barriers.

## Equitable access

Although ambulatory treatments for COVID-19 are widely available in the United States and are free to patients under the EUA, large disparities exist between high-income and low- to middle-income countries (LMICs) in the utilization of mAbs. The United States, Canada, and Europe dominate ∼80% of the global market.^50^ Low mAb use in LMICs is attributable to barriers in availability, affordability, and accessibility.^50^ Few clinical data exist describing mAb outcomes in LMICs. These agents are in limited supply, are manufactured exclusively by US companies, and are protected by intellectual property laws from commercial replication.^50^ The high cost of these agents, ∼$2,100 per treatment, and lack of collaborative agreements for approval between manufacturers and many LMIC governments precludes purchase.^50–53^ Nonexistent regulatory approval processes for biologic products further impedes mAb development in >50% of LMICs.^50^ LMICs lack healthcare facilities and providers to ensure appropriate transportation, storage, and parenteral administration.^50^ Limited testing capacities in LMICs inhibits timely connection to care for infected patients.

Oral antivirals offer several advantages for use in LMICs compared to mAbs. Their production requires only standard pharmaceutical manufacturing equipment used in the production of small-molecule drugs, in contrast to the specialized technology and enhanced sterility precautions necessary for mAbs.^50,54^ Manufacturing costs for oral antivirals are less costly than for mAbs,^55,56^ and no refrigeration is required, unlike mAbs and vaccines.^54^

Both oral antiviral manufacturers signed voluntary licensing agreements in late 2021 with the Medicines Patent Pool, a United Nations–backed public health organization working to increase access to and facilitate the development of life-saving medications in LMICs. This agreement facilitates affordable access by allowing additional production and distribution of products by generic manufacturers at steep discounts, estimated at $14 per course for molnupiravir.^57^ The Medicines Patent Pool will be permitted to grant nonexclusive sublicenses to any qualified manufacturer worldwide, which will enable the Pool to supply medication to >95 countries. Both parent companies agreed to not receive royalties on sales in low-income countries and to waive royalties on sales in all countries covered under the agreement while COVID-19 remains a public health emergency. Finally, since both oral antivirals are not currently under patent, countries not included in this licensing agreement have opportunities to produce generic products directly. As access to outpatient antivirals steadily improves in LMICs, we suggest that ASP-led implementation, tracking of utilization, and reporting of outcomes be undertaken there.

In conclusion, after COVID-19 vaccines, outpatient SARS-CoV-2 therapeutics represent the greatest scientific advancement in the pandemic response. Multiple options now exist for treatment of mild-to-moderate, early COVID-19 in high-risk patients, most of which have significant efficacy against hospitalization and death. Success in the real world depends on equitable access to testing, global production, and dissemination of cutting-edge therapies. However, regardless of the supply–demand calculus, optimization requires content expertise, education of patients and providers, electronic tools to reduce barriers to uptake, and effective use of outcomes data to inform therapeutic decisions and evaluate the impact of new variants. As we enter the post-omicron phase of the pandemic, in which the public is eager to move on and preventive measures are relaxed, we must increasingly rely on available therapeutic and prophylactic options to protect the most vulnerable populations. Antimicrobial stewardship programs possess the expertise, experience, and track record for leading outpatient therapeutic interventions. They can help transform COVID-19 into a predominantly outpatient illness, ideally achieved on a global scale.
